# Photocatalytic Oxidation of Low-Level Airborne 2-Propanol and Trichloroethylene over Titania Irradiated with Bulb-Type Light-Emitting Diodes

**DOI:** 10.3390/ma6010265

**Published:** 2013-01-18

**Authors:** Wan-Kuen Jo

**Affiliations:** Department of Environmental Engineering, Kyungpook National University, Daegu, 702-701, South Korea; E-Mail: wkjo@knu.ac.kr; Tel: +82-53-950-6584; Fax: +82-53-950-6584

**Keywords:** baking temperature, X-ray diffraction, photocatalytic decomposition, black-light lamp, operational conditions

## Abstract

This study examined the photocatalytic oxidation of gas-phase trichloroethylene (TCE) and 2-propanol, at indoor levels, over titanium dioxide (TiO_2_) irradiated with light-emitting diodes (LED) under different operational conditions. TiO_2_ powder baked at 450 °C exhibited the highest photocatalytic decomposition efficiency (PDE) for TCE, while all photocatalysts baked at different temperatures showed similar PDEs for 2-propanol. The average PDEs of TCE over a three hour period were four, four, five, and 51% for TiO_2_ powders baked at 150, 250, 350, and 450 °C, respectively. The average PDEs of 2-propanol were 95, 97, 98, and 96% for TiO_2_ powders baked at 150, 250, 350, and 450 °C, respectively. The ratio of anatase at 2θ = 25.2° to rutile at 2θ = 27.4° was lowest for the TiO_2_ powder baked at 450 °C. Although the LED-irradiated TiO_2_ system revealed lower PDEs of TCE and 2-propanol when compared to those of the eight watt, black-light lamp-irradiated TiO_2_ system, the results for the PDEs normalized to the energy consumption were reversed. Other operational parameters, such as relative humidity, input concentrations, flow rate, and feeding type were also found to influence the photocatalytic performance of the UV LED-irradiated TiO_2_ system when applied to the cleaning of TCE and 2-propanol at indoor air levels.

## 1. Introduction

Long-term exposure of building occupants to volatile organic compounds (VOCs) at even low levels has recently been of concern because it is closely associated with adverse health effects [1,2]. Many VOC sources such as building materials and consumer products have been identified in residential and industrial buildings [3,4]. VOCs including trichloroethylene (TCE) and 2-propanol are generally detected at higher concentrations in indoor environments than outdoor environments [5]. Moreover, most VOCs are toxic or potentially toxic to humans [6], necessitating the development of means of indoor VOC purification.

A traditional technology for purifying indoor VOCs is the adsorption process; however, this only transfers VOCs to another phase rather than oxidizing them into innocuous compounds such as water vapor and carbon dioxide [7]. Heterogeneous photocatalytic processes over semiconductors have recently been proposed as an alternative means for control of indoor air pollution [8]. Of all semiconductors, titanium dioxide (TiO_2_) has been the most popular catalyst utilized for oxidation of environmental organic pollutants due to its high photo-activity, excellent stability, and low cost [9]. The oxidation of VOCs occurs as a result of reactions with molecular oxygen or hydroxyl radicals and super-oxide ions formed after the initial production of highly reactive electrons and hole pairs when the TiO_2_ is ultraviolet (UV)-irradiated [10].

The photocatalytic process over TiO_2_ requires a UV-light source that exceeds the band-gap potential of 3.0 or 3.2 eV in its rutile or anatase crystalline phases, respectively [9]. To date, many studies that have investigated TiO_2_ powders for environmental applications [11,12,13] have used conventional UV lamps, such as black-light and mercury lamps, with major emissions. However, the use of light-emitting diodes (LEDs) as semiconductor light sources has several advantages over conventional light sources. Specifically, LEDs are more efficient at converting electricity into light due to high quantum yields close to unity that lead to low electricity consumption. Additionally, they have a long life, with a typical lifetime of 25,000 to 100,000 h [14]. In addition, LEDs provide a tunable, almost monochromatic light. These characteristics have prompted the application of LEDs to the photocatalytic decomposition of environmental pollutants instead of conventional lamps. A few researchers [15,16] have suggested the feasibility of the use of UV LEDs in photocatalytic applications for the efficient removal of certain hazardous pollutants. Specifically, Shie *et al*. [15] reported that UV LEDs had superior energy effectiveness, when compared to conventional UV lamps, for the photocatalytic decomposition of gaseous formaldehyde.

However, previous studies that used TiO_2_/LED systems have primarily dealt with concentrations substantially higher than typical indoor levels [5], although certain studies that used TiO_2_/conventional UV lamp systems have dealt with indoor levels of aromatic VOCs [17,18]. In most cases, VOC concentrations in residential buildings are much lower than sub-ppm [5]. The extrapolation of photocatalytic performance data collected at concentrations much higher than those in their intended application may not be appropriate. Consequently, the current study investigated the photocatalytic oxidation of gaseous TCE and 2-propanol, at indoor levels, over TiO_2_ irradiated with LEDs using an annular-type plug-flow reactor. These target compounds were selected based on their prevalence in indoor environments [5]. Since LEDs have narrower irradiation angles (equal to or less than 140^o^) than conventional cylindrical lamps, the reactor was constructed to allow uniform light distribution onto the surfaces of the TiO_2_. The present study also examined the effects of baking temperature (BT), light-source type (LST), relative humidity (RH), input concentrations (IC), flow rate (FR), and feeding type (FT).

## 2. Results and Discussion

### 2.1. Comparison of Baking Temperatures for Photocatalytic Decomposition Efficiencies (PDEs)

**Figure 1:**
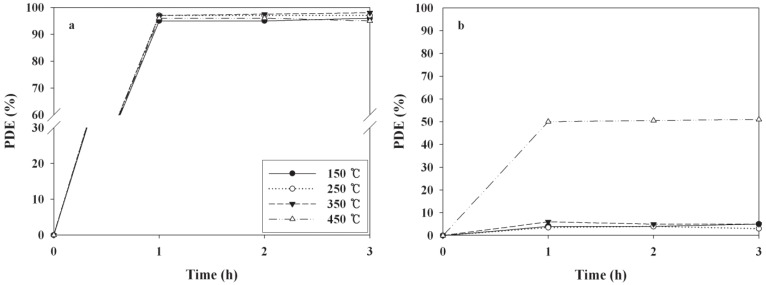
Time-series photocatalytic efficiencies (PDEs, %) of 2-propanol and gas-phase trichloroethylene (TCE) as determined at the outlet of the photocatalytic reactor, according to baking temperatures (150, 250, 350, or 450 °C).

**Figure 2:**
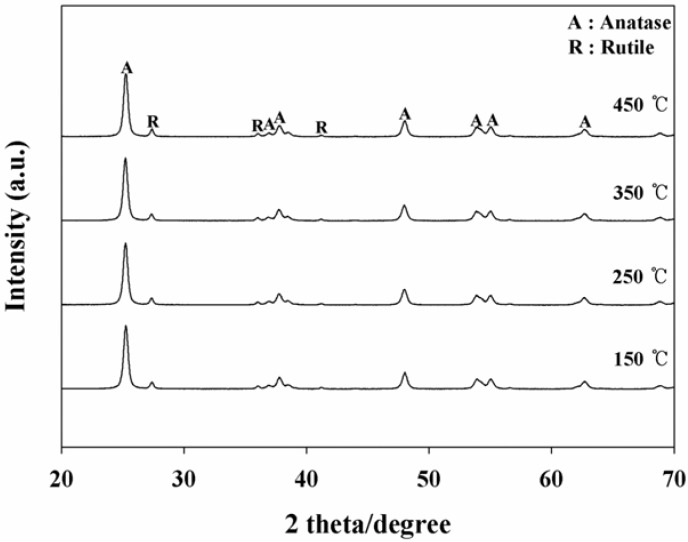
X-ray diffraction spectra of photocatalysts baked at four different temperatures (150, 250, 350, or 450 °C).

The effects of BT—applied for coating the as-prepared photocatalysts onto the Pyrex tube on the photocatalytic decomposition efficiencies (PDE) of 2-propanol and TCE—were investigated. Figure 1 shows the time-series PDEs of the two target compounds, according to BTs. The TiO_2_ powder baked at 450 °C exhibited the highest PDEs for TCE, while all photocatalysts baked at different temperatures showed similar PDEs for 2-propanol. The average PDEs of TCE over a three hour period were four, four, five, and 51% for the TiO_2_ powders baked at 150, 250, 350, and 450 °C, respectively. The average PDEs of 2-propanol were 95, 97, 98, and 96% for the TiO_2_ powders baked at 150, 250, 350, and 450 °C, respectively. The highest PDE for TCE obtained from the TiO_2_ powders baked at 450 °C was attributed to more transformation of the rutile phase TiO_2_ at this BT. This assertion is supported by the X-ray diffraction (XRD) results of the TiO_2_ powders baked at the four different temperatures (Figure 2). On the basis of the XRD results, the ratio of anatase at 2θ = 25.2° to rutile at 2θ = 27.4° was lowest for the TiO_2_ powder baked at 450 °C (Table 1), indicating more formation of the rutile phase when compared to TiO_2_ powders baked at the other temperatures. These findings are not consistent with those of a previous report, that anatase had a generally higher photocatalytic activity than rutile [7]. Instead, the low ratio found in the present study might result in less electron-hole recombination, elevating the photocatalytic performance of TiO_2_ for TCE decomposition. Similarly, Puddu *et al*. [19] reported that gaseous TCE degradation increased notably as the calcination temperature of anatase TiO_2_ increased from 300 to 600 ^o^C, while it decreased when the temperature was further increased to 700 and 800 °C. In addition, Znad and Kawase [18] reported that the decolorization rate of the Orange II increased by six times as the calcination temperature of the S-doped TiO_2_ increased from 100 to 550 °C. This was ascribed to gradual crystallization of the anatase TiO_2_ with increasing temperature, while further increases in the calcination temperature (to 700 °C) resulted in an obvious decrease in activity, which was ascribed to the increase in crystalline size. Meanwhile, surface area of TiO_2_ photocatalyst decreases as CT increases [19]. Therefore, a possible explanation for the highest PDE observed for the TiO_2_ calcined at the highest temperature is that suggests that the effect of gradual crystallization of the anatase TiO_2_ with increasing temperature would overweigh the effect of surface area on PDEs. Moreover, Nosaka *et al*. [19] found that the photocatalytic activity of N-doped TiO_2_, which was determined from the formation of acetone, was highest for the photocatalyst calcined at 350 °C within a calcination temperature range of 350−550 °C. Consequently, the effect of calcination temperature on photocatalytic activity likely depends on operational conditions, such as the type of photocatalyst and survey temperature range.

**Table 1:** The ratio of anatase at 2θ = 25.2° to rutile at 2θ = 27.4° according to baking temperature*.

Baking temperature (°C)	Ratio of anatase/rutile
150	8.8
250	8.5
350	8.8
450	7.7

* Ratio of anatase/rutile at room temperature, 2.3.

### 2.2. Comparison of Light Sources for PDEs

**Figure 3:**
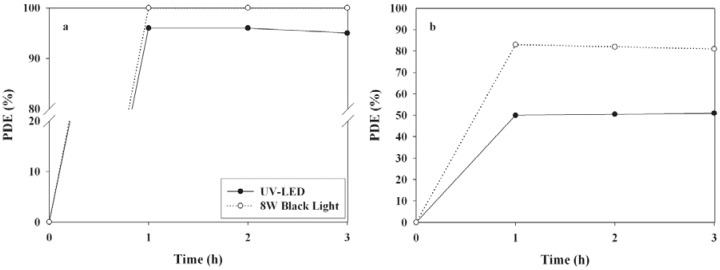
Time-series photocatalytic efficiencies (PDEs, %) of (**a**) 2-propanol; and (**b**) TCE as determined using TiO_2_ calcined at 450 °C according to light-source type (8 W black lamp and UV LEDs).

Figure 3 shows the time-series PDEs of 2-propanol and TCE determined using a conventional, eight watt, black-light lamp- and UV LED-irradiated TiO_2_ system over a three hour process. The conventional, eight watt, black-light lamp/TiO_2_ system exhibited a higher PDE than the UV LED/TiO_2_ system. The average PDEs of 2-propanol and TCE obtained from the three hour process of the eight watt black-light lamp/TiO_2_ system were ~100 and 82%, respectively, whereas those obtained from the three hour process of the UV LED/TiO_2_ system were 96 and 51%, respectively. TiO_2_ is a semiconductor photocatalyst with a band gap energy of 3.2 eV and this band gap energy is exceeded with photons of less than 385 nm [20,21]. Accordingly, both light sources have sufficient energy to promote electrons from the valence band to the conduction band of TiO_2_, as the eight watt black-light lamp and UV LEDs exhibit the maximum light intensity at 352 and 365 nm, respectively. Moreover, the light intensity of the eight watt black-light lamp (2.3 mW cm^−2^) was higher than that of the UV LEDs (0.98 mW cm^−2^). Therefore, these results indicate that the higher decomposition efficiency for the eight watt black-light lamp-irradiated TiO_2_ system was likely due to the lower wavelength (higher energy) and higher light intensity.

Moreover, the PDEs normalized to the energy consumption (% W^−1^) were calculated to evaluate the energy effectiveness of the two light sources. In contrast to the PDEs, the normalized PDEs obtained from the UV LED-irradiated TiO_2_ system were higher than those from the eight watt black-light lamp-irradiated TiO_2_ system. The normalized PDEs of 2-propanol and TCE for the UV LED-irradiated TiO_2_ system were 34 and 18% W^−1^, respectively, while those for the eight watt black-light lamp-irradiated TiO_2_ system were 13 and 10% W^−1^, respectively. These findings confirm that UV LEDs can be utilized as energy-efficient light sources for the photocatalytic decomposition of 2-propanol and TCE. Accordingly, when considering the energy efficiency of light sources, UV LEDs are recommended as light sources of the TiO_2_ photocatalytic system. However, it should be noted that conventional lamps are preferable when higher photocatalytic removal efficiencies are required, because the conventional lamp/photocatalytic systems exhibited higher PDEs efficiencies than the LED/photocatalytic systems.

### 2.3. Effects of Operational Conditions on PDEs

**Figure 4:**
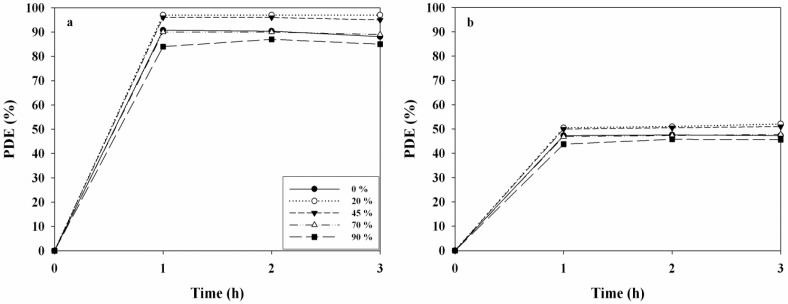
Time-series photocatalytic efficiencies (PDEs, %) of (**a**) 2-propanol; and (**b**) TCE as determined using TiO_2_ calcined at 450 ^o^C according to relative humidity (20, 45, 70, and 90%).

The effects of operational conditions of the UV LED-irradiated TiO_2_ system on PDEs of 2-propanol and TCE were investigated. The operational conditions were determined by varying the RH, ICs, FR, and FT. Figure 4 reveals the PDEs of 2-propanol and TCE obtained from the photocatalytic system according to RH. The PDE of 2-propanol decreased from 97% to 85%, as the RH increased from 20% to 90%. With respect to high RH conditions, excessive water molecules could preferably occupy active sites on the photocatalyst surface, causing a decrease in the reaction rate. Similarly, Pengyi *et al*. [22] reported that the toluene decomposition efficiency decreased as RH increased from 55% to 95% , when TiO_2_ was used as a photocatalyst under similar experimental conditions (0.02–0.4 ppm). However, the PDEs of TCE did not exhibit a distinct trend as the RH increased. The maximum PDE of TCE was observed at an RH of 45%, whereas the PDEs of TCE determined under the other RH conditions were close to zero. When TCE was considered, the drop in PDE at low RH values was attributed to a decrease in the hydroxyl radical population on the catalyst surface, whereas the decrease in the PDE at high RHs was a result of competitive adsorption between water and the contaminant on the catalyst surface [23]. Consequently, the two target compounds (2-propanol and TCE) revealed different RH dependence on PDE, indicating that photocatalytic kinetics associated with RH conditions would differ between the two target compounds. In addition, the PDEs of 2-propanol and TCE determined under dried conditions were lower than those determined at the RH of 20%, which was ascribed to less OH radical formation in the absence of water vapor [24].

**Figure 5:**
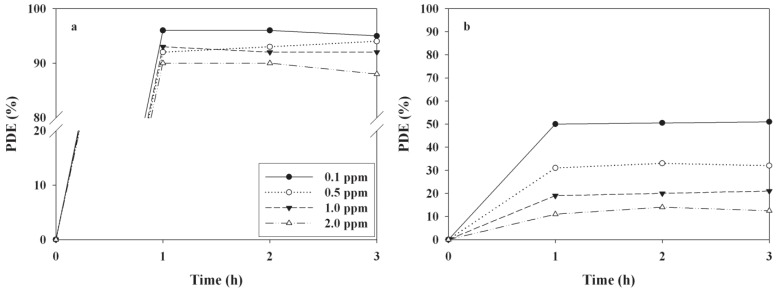
Time-series photocatalytic efficiencies (PDEs, %) of (**a**) 2-propanol; and (**b**) TCE as determined at the outlet of the photocatalytic reactor according to input concentrations (0.1, 0.5, 1.0, and 2.0 ppm).

The PDEs of the two target compounds obtained from the UV LED-irradiated TiO_2_ photocatalytic system according to IC are presented in Figure 5. In most cases, the PDEs for the target compounds exhibited a decreasing trend with increasing IC. The average decomposition efficiencies for 2-propanol and TCE over a three hour process decreased from 96% to 89% and 51% to 13%, respectively, as the IC increased from 0.1 to 2.0 ppm. These findings are consistent with those reported in other studies [22,25] that used conventional lamp-irradiated TiO_2_ systems. The IC dependence was ascribed to adsorptive competition between molecules of 2-propanol and TCE for the active adsorption sites on the TiO_2_ surface. For higher ICs, the active adsorption sites on the photocatalyst surface might be more limited for adsorption of molecules of target compounds. This decreasing pattern in PDEs with IC is also associated with the Langmuir–Hinshelwood (LH) adsorption isotherm model, which has most frequently been utilized to link the photocatalytic reaction rate of VOCs to their ICs [25]. On the basis of the LH model, the decreasing pattern in PDEs implies that the concentration range used in the present study corresponds to the intermediate regime, in which the photocatalytic reaction rate increased slowly but the PDE decreased. For the high IC regime, in which the photocatalytic oxidation is independent of IC (zero-order kinetics) the PDE would be reduced. This assertion is supported by the finding that in contrast to the degradation efficiencies, the photocatalytic oxidation rates of 2-propanol and TCE, which were calculated by using the following equation, increased as the IC increased (Table 2):

*r = f(C_in_ – C_out_)Q/A* (1)

where *C_in_* and *C_out_* present the input and output concentrations (ppm), respectively; *Q* represents the air flow rate (m^3^ s^−1^); *A* represents the photocatalyst-coated area (m^2^); and *f* presents the conversion factor (40.9 μ-mole m^−3^ ppm^−1^). The data represented in Table 2 is consistent with that of a previous study [26].

**Table 2:** Reaction rate (μ-mole m^−2^ s^−1^) of 2-propanol and TCE according to initial concentration (IC)

Compound	IC (ppm)			
	0.1	0.5	1.0	2.0
2-Propanol	1.2 × 10^−3^	4.1 × 10^−3^	5.7 × 10^−3^	8.7 × 10^−3^
TCE	1.0 × 10^−3^	2.1 × 10^−3^	3.3 × 10^−3^	5.2 × 10^−3^

**Figure 6:**
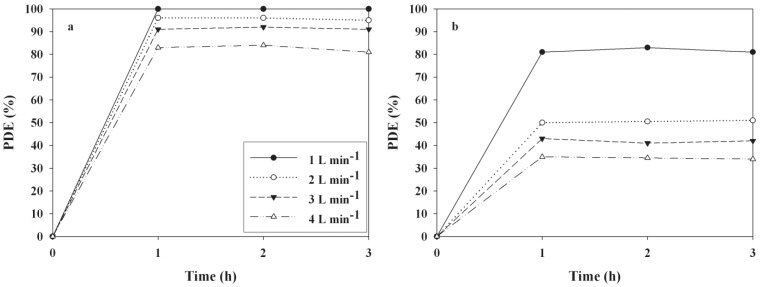
Time-series photocatalytic efficiencies (PDEs, %) of (**a**) 2-propanol; and (**b**) TCE as determined using TiO_2_ calcined at 450 ^o^C according to flow rates (1.0, 2.0, 3.0, and 4.0 L min^−1^).

Figure 6 exhibits the PDEs of 2-propanol and TCE as determined by the UV LED-irradiated TiO_2_ photocatalytic system at four different FRs. The PDEs of both compounds decreased as the FR increased. As the FR increased from 1.0 to 2.0 L min^−1^, the average PDEs of 2-propanol and TCE obtained from the three hour photocatalytic process decreased from ~100 to 83% and 82 to 35%, respectively. The lower PDEs at the high FRs were most likely due to an insufficient reactor retention time for these pollutants to transfer from the gas phase to the catalyst surface. In fact, the retention times in the present study, which were calculated by dividing the reactor volume by FR, were 11, 5.5, 2.75, 1.38, and 0.69 s for FRs of 1.0, 2.0, 3.0, and 4.0 L min^−1^ respectively. However, the face velocities, which were estimated by dividing volumetric FR by the cross-sectional area of the photocatalytic reactor, decreased as the retention times increased. The mass transfer of bulk compounds to the surface of the photocatalyst is closely associated with face velocity, which is an important factor that influences PDEs [26,27,28]. In a low face velocity regime, the mass transfer tends to increase as the face velocity increases, which elevates photocatalytic reaction rates [26]. In contrast, the PDEs obtained from the current study decreased as the face velocities increased. Therefore, the lower PDEs of 2-propanol and TCE for shorter retention times (higher face velocities) are ascribed to an insufficient reaction time in the photocatalytic reactor for the photocatalytic oxidation of these compounds, but not the mass transfer effect.

**Figure 7:**
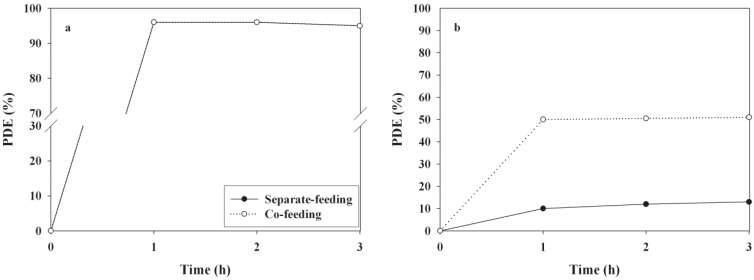
Time-series photocatalytic efficiencies (PDEs, %) of (**a**) 2-propanol; and (**b**) TCE as determined using TiO_2_ calcined at 450 °C according to feeding type (separate- and co-feeding).

The effect of FT (separate and co-feeding) on PDEs of 2-propanol and TCE as determined via the UV LED-irradiated TiO_2_ photocatalytic system were investigated. Figure 7 illustrates the PDEs of the measured compounds with respect to the two FTs. The analysis of variance exhibited that the PDEs of TCE were significantly elevated with co-feeding when compared to separate feeding (*p* < 0.05), whereas those of 2-propanol did not change significantly. The average TCE degradation efficiency during co-feeding was 51%, whereas it was approximately 12% during separate feeding. The increased degradation efficiency of TCE during co-feeding was likely due to hydroxyl radicals produced during the photodegradation of 2-propanol, because the hydroxyl radicals were considered the major active species in the photocatalytic oxidation of organic pollutants [29]. In contrast, other studies [30,31,32,33,34] reported that chlorinated olefins, TCE, perchloroethylene (PCE) and 1,1,3-trichloropropene (TCP), promoted the PDEs of aromatic VOCs. Specifically, Sauer *et al*. [33] demonstrated that when PCE or TCP was fed with toluene, the conversion of the latter was raised to 100%. Previous studies [] suggested that the function of the added sensitizers (TCE, PCE, and TCP) is to provide the radicals required to initiate chain-propagated destruction of the pollutant. In addition, Young *et al*. [34] proposed a specific mechanism involving a new activation path for toluene, *i.e.*, a chain transfer from the active chlorine in the chlorocarbon oxidation chain. Accordingly, these results suggest that PDE dependence on the FT of gas-phase pollutants varies with the types of pollutants and photocatalytic degradation pathways.

## 3. Experimental Section

**Figure 8:**
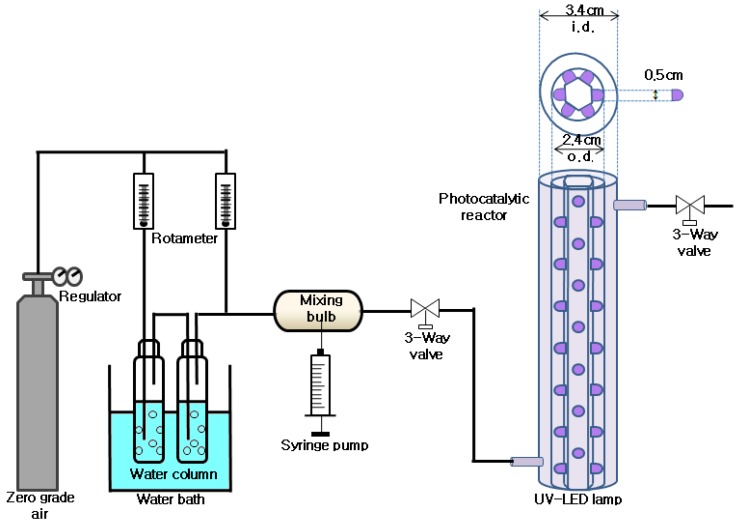
Schematic diagram of experimental set-up.

The photocatalytic reactor consisted of a cylindrical Pyrex tube (inner diameter, 4.0 cm) coated on the inner surface with a thin film of TiO_2_ photocatalyst (Degussa P-25, Japan Aerosil, Mie, Japan) and another Pyrex tube with a smaller outside diameter (2.5 cm) (Figure 8). A cylindrical, conventional UV lamp or bulb-type LEDs were inserted inside the smaller Pyrex tube. The LEDs were supported by a hexahedral tube made of Teflon. The gas flowed through the annular region between the two Pyrex tubes. This design was well adapted to the research environment because it provided a well-characterized reactive catalyst surface along the length of the reactor body and allowed uniform light distribution. The application of a thin, uniform TiO_2_ coating on the inside of the reactor tubes was essential; thus, the Pyrex surface was coated using TiO_2_ slurry. For the coating process of these photocatalysts, ground photocatalyst powders were added to 0.1 M ethylenediaminotetraacetic acid solution. This mixture was diluted by slowly adding deionized water and Triton X-100 in turn, and the resultant sol was pasted onto the inner wall of the reactor. The pasted reactor was dried in an oven at 100 °C for 0.5 h and then calcined in a furnace at a specified temperature (150, 250, 350, or 450 °C) for 0.5 h. The coated reactor was dried for an hour at room temperature and then baked for 30 minutes at a specified temperature. The humidity levels were then adjusted by passing zero-grade air through a charcoal filter, followed by a humidification device in a water bath. The RH was measured immediately in front of the reactor inlet using a humidity meter (Thermo Recorder TR-72S, T & D Co., Tokyo, Japan). FR measurements were carried out using rotameters with a range of 0–10 L min^−1^. The air stream was then externally heated to facilitate vaporization of the injected target compounds into a mixing chamber via a syringe pump (Model 210, KdScientific Inc., Holliston, MA, USA). Finally, the air stream was fed into the annular region between the two Pyrex tubes. A standard solution of the target compounds prepared in methanol (HPLC grade, Sigma-Aldrich, St. Louis, MO, USA) was infused into the syringe of the syringe pump.

The photocatalytic degradation efficiency of the TCE and 2-propanol were investigated under various experimental conditions by varying BT, LST, RH, IC, FR, and FT. The BTs for coating the TiO_2_ onto the reactor wall were 150, 250, 350, or 450 °C. The compositional characteristics of the photocatalysts baked at different temperatures were investigated using an X-ray diffraction (XRD) meter operated at 40 kV and 100 mA (D/max-2500 diffractometer, Rigaku Inc., Tokyo, Japan). The UV radiation was supplied by UV LEDs (MS-L510UV120, Moksan Electric Co., Seoul, Korea) or an eight watt fluorescent black light lamp (F8T5/BLB-352 nm, Sankyo Denki Co., Osaka, Japan). The RH range for these experiments was 20%–90% (20, 45, 70, and 90%) to cover both dried and humidified environments. The ICs of 2-propanol and TCE varied from 0.1 to 2.0 ppm (0.1, 0.5, 1.0, and 2.0 ppm). The range of FRs investigated was 1.0 to 4.0 L min^−1^ (1.0, 2.0, 3.0, and 4.0 L min^−1^). Two target compounds (2-propanol and TCE) were fed into the photocatalytic reactor separately or together.

Gas-phase compounds in the air stream were measured at the inlet and outlet of the photocatalytic reactor. Gas samples were collected by filling an evacuated five liter Tedlar bag at a constant FR. Air from this bag was then drawn through a 0.64-cm-o.d. and 18-cm-length SS sorbent trap containing 0.5 grams of Tenax TA using a constant flow-sampling pump (Model I.H, A.P. Buck Inc., Orlando, FL, USA). Sampling times varied from one to five minutes depending on the FR. All samples were taken at ambient room temperature. The gaseous compounds collected on the Tenax TA trap were analyzed by coupling a thermal desorber (TD 200, Donam Co., Tustin, South Korea) to a gas chromatograph (GC, 7890, Agilent Inc., Santa Clara, CA, USA), with a flame ionization detector (FID) using a fused silica column (SPB-5, Supelco Co., Bellefonte, PA, USA). Next, the adsorbent (Tenax TA) trap was thermally desorbed at 250 °C for 10 min, and the target compounds were cryofocussed at −30 °C on a cryo trap (15.2-cm-length, 0.32-cm-o.d. tube packed with glass beads). The cold trap was rapidly heated to 250 °C and then flushed to transfer the target compounds to a GC. The initial oven temperature was set to 35 °C for five min, and ramped at 4 °C min^−1^ to 200 °C, where it was held for five minutes. In the present study, the target VOCs were identified by their retention times using GC/FID analysis. Quantitative analysis of the target compounds was conducted using calibration curves based on a minimum of five concentrations.

The quality assurance/quality control program included laboratory blank traps and spiked samples. At the beginning of the day, a laboratory blank trap was analyzed to check for any trap contamination; however, no contamination was identified. An external standard was analyzed daily to check the quantitative response. When the quantitative response differed from that predicted by the specified calibration equation by more than 20%, a new calibration equation was determined. The method detection limits for 2-propanol and TCE were 0.003 and 0.01 ppm, respectively.

## 4. Conclusions

This study explored the photocatalytic oxidation of gaseous TCE and 2-propanol at indoor levels over TiO_2_ irradiated with LEDs using an annular-type plug-flow reactor. This investigation was performed under a range of operational conditions. The PDEs of TCE varied with BT of TiO_2_ powder, while the PDEs of 2-propanol were similar, regardless of BT. These findings were associated with the ratios of anatase to the rutile crystal phase of TiO_2_ photocatalysts baked at different temperatures, which were determined using XRD results. The eight watt black-light lamp-irradiated TiO_2_ system exhibited a higher decomposition efficiency than the UV LED-irradiated TiO_2_ system, which was attributed to the lower wavelength (higher energy) and higher light intensity. However, the results for the PDEs normalized to the energy consumption were reversed, indicating that, when considering energy efficiency of light sources, UV LEDs are recommended as light sources for the TiO_2_ photocatalytic system. It was also highlighted that other operational parameters, such as RH, ICs, FR, and FT should be considered for the optimum application of the UV LED-irradiated TiO_2_ system to purification of indoor air TCE and 2-propanol.
